# ctDNA-based detection of molecular residual disease in stage I-III non-small cell lung cancer patients treated with definitive radiotherapy

**DOI:** 10.3389/fonc.2023.1253629

**Published:** 2023-09-19

**Authors:** Emily S. Lebow, Narek Shaverdian, Jordan E. Eichholz, Leah B. Kratochvil, Megan McCune, Yonina R. Murciano-Goroff, Justin Jee, Juliana Eng, Jamie E. Chaft, Mark G. Kris, Ekaterina Kalashnikova, Jordan Feeney, Carly Bess Scalise, Sumedha Sudhaman, Charuta C. Palsuledesai, Meenakshi Malhotra, Michael Krainock, Himanshu Sethi, Alexey Aleshin, Minetta C. Liu, Annemarie F. Shepherd, Abraham J. Wu, Charles B. Simone, Daphna Y. Gelblum, Kaylie A. Johnson, Charles M. Rudin, Daniel R. Gomez, Pedram Razavi, Jorge S. Reis-Filho, James M. Isbell, Bob T. Li, Andreas Rimner

**Affiliations:** ^1^ Memorial Sloan Kettering Cancer Center, New York, NY, United States; ^2^ The University of Pennsylvania, Philadelphia, Pennsylvania; ^3^ Weill Cornell Medicine, Cornell University, New York, NY, United States; ^4^ Natera, Inc., Austin, TX, United States

**Keywords:** circulating tumor DNA (ctDNA), tumor-informed, molecular residual disease (MRD), non-small cell lung cancer (NSCLC), definitive radiation, prognostic biomarker

## Abstract

**Background:**

Sensitive and reliable biomarkers for early detection of recurrence are needed to improve post-definitive radiation risk stratification, disease management, and outcomes for patients with unresectable early-stage or locally advanced non-small cell lung cancer (NSCLC) who are treated with definitive radiation therapy (RT). This prospective, multistate single-center, cohort study investigated the association of circulating tumor DNA (ctDNA) status with recurrence in patients with unresectable stage I-III NSCLC who underwent definitive RT.

**Methods:**

A total of 70 serial plasma samples from 17 NSCLC patients were collected before, during, and after treatment. A personalized, tumor-informed ctDNA assay was used to track a set of up to 16 somatic, single nucleotide variants in the associated patient’s plasma samples.

**Results:**

Pre-treatment ctDNA detection rate was 82% (14/17) and varied based on histology and stage. ctDNA was detected in 35% (6/17) of patients at the first post-RT timepoint (median of 1.66 months following the completion of RT), all of whom subsequently developed clinical progression. At this first post-RT time point, patients with ctDNA-positivity had significantly worse progression-free survival (PFS) [hazard ratio (HR): 24.2, p=0.004], and ctDNA-positivity was the only significant prognostic factor associated with PFS (HR: 13.4, p=0.02) in a multivariate analysis. All patients who developed clinical recurrence had detectable ctDNA with an average lead time over radiographic progression of 5.4 months, and post-RT ctDNA positivity was significantly associated with poor PFS (p<0.0001).

**Conclusion:**

Personalized, longitudinal ctDNA monitoring can detect recurrence early in patients with unresectable NSCLC patients undergoing curative radiation and potentially risk-stratify patients who might benefit most from treatment intensification.

## Introduction

Non-small cell lung cancer (NSCLC) accounts for 85% of lung cancer-related diagnoses and deaths (1). The current standard-of-care for patients with inoperable NSCLC is definitive curative radiotherapy (RT) including stereotactic body radiotherapy (SBRT) for early-stage disease and concurrent chemoradiation (CRT) followed by durvalumab for locally-advanced disease (2).

For early-stage disease, SBRT has shown excellent long-term primary tumor control rates, with nodal and distant recurrences representing the most common failure pattern (3). In patients with locally advanced NSCLC, consolidation durvalumab significantly improves the progression-free survival (PFS) compared to CRT alone, yet in-field recurrence and distant metastases present a challenge post-CRT and durvalumab. Thus, careful long-term surveillance is necessary for early detection of recurrence before the onset of disease-related symptoms and at a time when therapy might provide greater clinical benefit. The current surveillance protocol includes computed tomography (CT) of the chest every 3 months for 2 years, every 6 months during years 3 and 4, and annually thereafter (2). Radiographic surveillance is associated with several challenges such as low sensitivity, detection of macroscopic disease, and difficulties in interpretation of results due to post-treatment effects, such as inflammatory changes, radiation fibrosis, or reactive lymph nodes in cases with local recurrences (4). Therefore, a need exists for a sensitive, blood-based biomarker for early detection of molecular residual disease (MRD), post-definitive therapy.

Circulating tumor DNA (ctDNA) has emerged as a prognostic biomarker to assess MRD and predict recurrence (5, 6). In this study, we assessed the prognostic value of a tumor-informed ctDNA assay for longitudinal monitoring of patients with stage I-III NSCLC undergoing definitive radiotherapy to detect recurrence and identify patients who might benefit from intensification of systemic therapy.

## Methods

### Subjects and study design

All patients had a pathologically confirmed diagnosis of lung cancer. Blood samples (n=70) serially collected (before and after SBRT as well as before, during, and after conventional RT with/without concurrent systemic therapy and adjuvant durvalumab) from a prospective clinical cohort of patients (N=17) with stage I-III NSCLC diagnosed between 2017 and 2020 were used for ctDNA analysis. All patients were staged according to American Joint Committee on Cancer (AJCC) 8^th^ Edition. Patients with stage I disease were treated with SBRT in 4 or 5 fractions (10 – 12 Gy per fraction). Patients with stage II and stage III disease were treated with conventional fractionation (2 Gy per fraction) with or without concurrent and adjuvant systemic therapy. Post-treatment plasma was collected generally concurrently with the standard-of-care imaging at the discretion of the treating clinician. Light, moderate, and heavy smokers were defined as <20 packs/year, 20-40 packs/year, and >40 packs/year, respectively. The longitudinal setting was defined as serial ctDNA testing of patients after discontinuation of RT (during systemic therapy, if given, and during surveillance), wherein patients had sample collection for ctDNA tests at regular intervals or as determined by the treating physician. This study was approved by the Memorial Sloan Kettering Institutional Review Board. The study was conducted in accordance with the principles of the Declaration of Helsinki 2013. All patients provided informed consent.

### Personalized ctDNA assay workflow

Personalized, tumor-informed ctDNA assays were designed for all patients as previously described (7). Briefly, a set of 16 high-confidence, patient-specific, somatic, clonal single nucleotide variants (SNVs) were selected for multiplex polymerase chain reaction (mPCR) testing from whole-exome sequencing of formalin-fixed paraffin-embedded (FFPE) tumor tissue and matched normal blood samples. The mPCR primers targeting the patient-specific SNVs were designed, synthesized, and used for tracking ctDNA in patients’ longitudinal plasma samples. Plasma samples were considered ctDNA-positive when at least 2 SNVs were detected above a predefined confidence threshold. ctDNA concentration was expressed as mean tumor molecules (MTM)/mL of plasma.

### Statistical analysis

Fisher’s exact test was used to evaluate the statistical significance of the association between ctDNA detection rates at baseline and categorical variables. Using the Kaplan-Meier method, PFS was assessed as the primary outcome between the date of RT initiation and clinical recurrence using post-RT ctDNA status for patient stratification. Log-rank test or Cox proportional hazards model was used for comparing two survival distributions with p ≤ 0.05 being considered significant. Statistical analyses were carried out in STATA v16.1.

## Results

Patient demographics, baseline characteristics, and treatment regimens are presented in **Table 1**. Patients were followed for a median of 26 months (range: 4-54). ctDNA assays were successfully designed for all patients.

**Table 1:** Patient demographics, baseline characteristics, treatment regimen, and outcome at the last follow-up.

Patient characteristics	Number of patients (%)
Stage	
I/II	7 (41.2)
III	10 (58.8)
Histology	
Squamous cell carcinoma	9 (52.9)
Adenocarcinoma	8 (47.1)
Sex	
Female	11 (64.7)
Male	6 (35.3)
Smoking status	
Light	4 (23.5)
Moderate	6 (35.3)
Heavy	6 (35.3)
Unknown	1 (5.9)
Radiotherapy	
SBRT	5 (29.4)
Conventional RT	12 (70.6)
Systemic chemotherapy	10 (58.8)
No systemic chemotherapy	7 (41.2)
Adjuvant Durvalumab	5 (29.4)
No adjuvant Durvalumab	12 (70.6)
Recurrence	
Yes	9 (52.9)
No	8 (47.1)
Deceased by 10/26/2021	8 (47.1)
Alive on 10/26/2021	9 (52.9)

SBRT, stereotactic body radiotherapy; ICI, RT, radiotherapy.

### ctDNA detection at baseline and association with PFS

**Figure 1:**
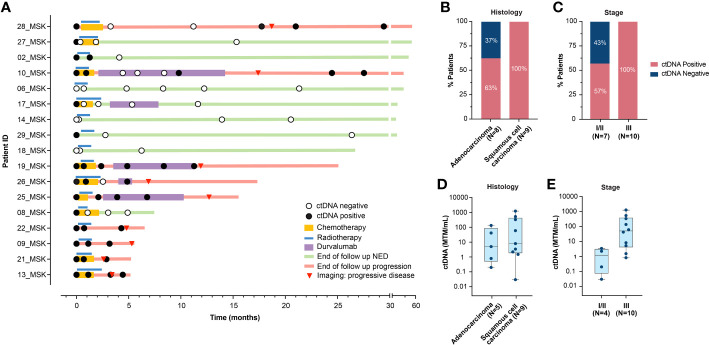
**(A)** Overview plot depicting treatment regimen, longitudinal ctDNA analysis and clinical outcomes for each patient in the cohort. **(B, C)**. Pre-RT ctDNA detection rate based on **(B)** histology, and **(C)** stage. **(D, E)**. Pre-RT ctDNA levels (MTM/mL) in 14 patients with detectable ctDNA based on **(D)** histology, and **(E)** stage. RT, radiotherapy; ctDNA, circulating tumor DNA; NED, no evidence of disease; MTM, mean tumor molecules.

At baseline (pre-RT time point), the ctDNA detection rate was 82% (14/17; **Figure 1A**) and varied based on histology and stage (**Figures 1B–E**). All patients with squamous cell carcinoma (9/9) were ctDNA-positive, whereas 63% (5/8) of patients with adenocarcinoma harbored detectable ctDNA (**Figure 1B**). All patients (10/10) with stage III disease were ctDNA-positive (**Figure 1C**) and presented with higher ctDNA levels, compared to patients with stage I/II disease (4/7; 57%) (**Figure 1E**). All patients with baseline ctDNA-negativity remained progression-free.

### Association of post-RT ctDNA status with PFS

**Figure 2:**
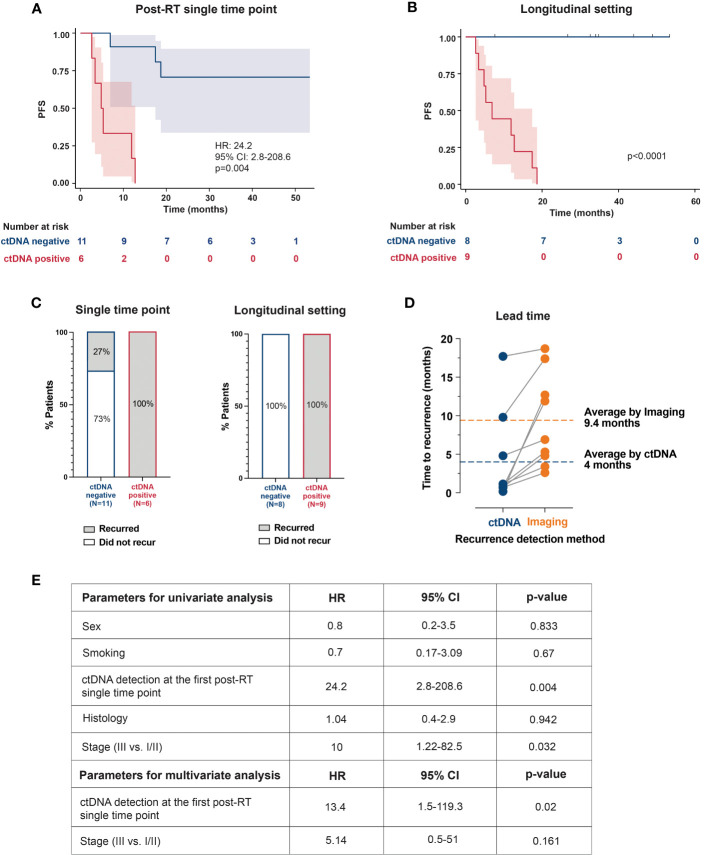
Kaplan–Meier plots representing the association of ctDNA positivity with PFS: **(A)** at the first available post-RT timepoint **(B)** and in longitudinal setting **(C)** The bar plots show recurrence rates in ctDNA-positive and ctDNA-negative patients at a single timepoint and longitudinally. **(D)** Comparison between ctDNA and radiographic imaging. Lead time indicates the number of months by which the ctDNA detected molecular recurrence ahead of radiological progression. **(E)** Univariate and multivariate analyses of prognostic factors and their association with PFS. RT, radiotherapy; PFS, progression-free survival; HR, hazard ratio; CI, confidence interval.

ctDNA detection rate at the first post-RT timepoint, collected at a median of 1.66 months (range: 0.4-13.7) was 35.3% (6/17), with all ctDNA-positive patients showing confirmed clinical progression. Additional three patients with transient ctDNA clearance eventually recurred. Compared to ctDNA-negativity at the first post-RT timepoint, patients with ctDNA-positivity had significantly worse PFS [hazard ratio (HR): 24.2, 95% confidence interval (CI): 2.8-208.6, p=0.004; **Figure 2A**].

In the longitudinal setting with serial ctDNA testing, post-RT ctDNA-positivity was associated with shorter PFS (p<0.0001; **Figure 2B**). Furthermore, all patients with confirmed clinical progression (9/17, 53%) were ctDNA-positive (sensitivity=100%; 9/9), and all ctDNA-negative patients remained progression-free (specificity=100%; 8/8; **Figure 2C**). Notably, ctDNA-based MRD detection had an average lead-time of 5.4 months over radiographic progression (**Figure 2D**). **Supplementary Figure 1** highlights clinical courses of 3 patients, depicting ctDNA dynamics during and after definitive RT and their correlation with radiographic response/progression.

In both univariate and multivariate analysis, ctDNA-positivity at the first post-RT timepoint was the strongest prognostic factor associated with PFS (HR: 24.2, p=0.004, and HR: 13.4, p=0.02), followed by stage (HR: 10, p=0.032; univariate analysis; **Figure 2E**).

## Discussion

Definitive radiation is the standard-of-care for patients with inoperable localized lung cancer. Our study demonstrates that tumor-informed ctDNA monitoring is an effective tool to detect MRD among patients treated with definitive RT. ctDNA monitoring preceded clinical recurrence by a median of 5.4 months, providing a critical window for early therapeutic intervention.

ctDNA monitoring is a promising technology to personalize therapy selection among patients with localized lung cancer. In the early-stage setting, the determination of adjuvant therapy after definitive RT is based on high-risk clinical and pathologic features (2). We observed that all patients with detectable MRD developed clinical recurrence, suggesting utilization of ctDNA may identify a group of patients at high risk of relapse who are likely to derive benefit from adjuvant therapy. In the locally advanced setting, one year of adjuvant durvalumab is standard-of-care for patients following definitive chemoradiation. MRD monitoring may allow for personalization of adjuvant therapy by identifying patients benefiting from consolidative durvalumab versus those who may benefit from an alternative approach (6). For example, in our cohort, patients who failed to clear ctDNA while receiving adjuvant durvalumab developed clinical recurrence. It is possible this cohort would have benefited from an alternative or intensified systemic therapy. An ongoing study (NCT04585490) evaluating personalized escalation of therapy for patients with locally advanced lung cancer treated with chemoradiation therapy will be an important contribution to patient care and demonstration of the clinical utility of ctDNA-based MRD analysis.

MRD monitoring may also identify patients with a low risk of recurrence for whom adjuvant therapy could be de-intensified. For example, among patients receiving adjuvant durvalumab in the PACIFIC trial, 19% who received placebo remained disease-free at 5 years, suggesting there are patients who do not derive clinical benefit but are exposed to toxicity of one year of adjuvant durvalumab (8). In a study by Monding et al, one patient with undetectable ctDNA died from pneumonitis related to immune checkpoint inhibition, highlighting the importance of identifying patients most likely to benefit from a therapy which poses a risk of high-grade toxicities (6).

Other studies in locally-advanced NSCLC utilizing different ctDNA technologies have demonstrated the utility of MRD detection at first timepoint (9), 1-month (10), or within 2 weeks to 4 months (11) of post-definitive treatment to be prognostic of clinical outcomes. In our study, ctDNA-positive patients at first post-RT timepoint were 24 times more likely to experience disease progression. Nonetheless, sensitivity was improved with longitudinal monitoring, which has been reported in lung cancer (9, 12) and other solid tumors (7, 13, 14).

In our cohort, we observed a baseline (pre-RT) ctDNA detection rate of 82% which was associated with stage and histology, consistent with prior analyses (6, 10–12). ctDNA detection is challenging in low-volume disease with limited ctDNA shedding, and further efforts are required to optimize detection in this patient population; however, it is important to note that the patients with baseline ctDNA-negativity had favorable outcomes.

Although blood samples were collected prospectively in this study, the correlation between ctDNA status and PFS was analyzed retrospectively, which precluded real-time assessment of risk of progression based on ctDNA status of patient at a given time point. Additionally, our study is limited by the small cohort size, heterogeneous disease stages and treatment regimens, and limited clinical follow-up for some patients. Nonetheless, our study demonstrated high sensitivity and specificity for ctDNA with for detection of recurrence with serial monitoring after completion of RT. Currently, the determination of adjuvant therapy in early-stage NSCLC patients after definitive RT is based on the presence of high-risk pathologic features (poorly differentiated tumor, vascular invasion, and visceral pleural involvement). However, our results highlight the potential utility of a personalized and tumor-informed ctDNA testing approach to risk-stratify patients for treatment decision-making. Prospective studies with larger cohorts are warranted to establish the clinical utility of ctDNA, particularly to determine the optimal interval for ctDNA testing, to validate the prognostic performance of longitudinal ctDNA monitoring, and to evaluate the benefits and risks of ctDNA-guided adjuvant treatment decision-making in NSCLC patients receiving curative RT.

## Data availability statement

The authors declare that all relevant data used to conduct the analyses are available within the article. To protect the privacy and confidentiality of patients in this study, clinical data cannot be made publicly available.

## Ethics statement

The studies involving humans were approved by Memorial Sloan Kettering Cancer Center IRB. The studies were conducted in accordance with the local legislation and institutional requirements. The participants provided their written informed consent to participate in this study.

## Author contributions

EL: Conceptualization, Data curation, Investigation, Methodology, Visualization, Writing – original draft, Writing – review & editing. NS: Conceptualization, Writing – review & editing. JE: Conceptualization, Data curation, Writing – review & editing. LK: Data curation, Writing – review & editing. MMc: Data curation, Writing – review & editing. YM-G: Conceptualization, Writing – review & editing. JJ: Conceptualization, Writing – review & editing. JE: Conceptualization, Writing – review & editing. JC: Conceptualization, Writing – review & editing. MGK: Conceptualization, Writing – review & editing. EK: Conceptualization, Data curation, Formal Analysis, Methodology, Project administration, Visualization, Writing – original draft, Writing – review & editing. JF: Writing – review & editing, Data curation, Formal Analysis, Investigation, Methodology, Visualization. CBS: Writing – review & editing, Data curation, Formal Analysis, Investigation, Methodology. SS: Writing – review & editing, Conceptualization, Data curation, Formal Analysis, Methodology. CCP: Conceptualization, Data curation, Formal Analysis, Methodology, Writing – original draft, Writing – review & editing. MMa: Writing – review & editing, Conceptualization, Data curation, Formal Analysis, Methodology. MGK: Writing – review & editing, Conceptualization, Data curation, Formal Analysis, Methodology. HS: Methodology, Writing – review & editing, Conceptualization, Data curation, Formal Analysis. AA: Conceptualization, Data curation, Formal Analysis, Methodology, Writing – review & editing. MCL: Conceptualization, Data curation, Formal Analysis, Methodology, Writing – review & editing. AS: Conceptualization, Writing – review & editing. AW: Conceptualization, Writing – review & editing. CSi: Conceptualization, Writing – review & editing. DG: Conceptualization, Writing – review & editing. KJ: Conceptualization, Project administration, Writing – review & editing. CR: Conceptualization, Writing – review & editing. DG: Concepualization, Writing – review & editing. PR: Conceptualization, Writing – review & editing. JR: Conceptualization, Writing – review & editing. JI: Conceptualization, Writing – review & editing. BL: Conceptualization, Investigation, Methodology, Visualization, Writing – review & editing. AR: Conceptualization, Data curation, Formal Analysis, Investigation, Methodology, Resources, Supervision, Visualization, Writing – original draft, Writing – review & editing. EL, EK, HS, and AR had full access to all the data in the study and takes responsibility for the integrity of the data and the accuracy of the data analysis.

## Supplementary material

Supplementary Figure 1 Patient-specific changes in ctDNA levels in response to treatment and radiographic imaging data. **(A)** Patient 17_MSK: ctDNA clearance indicated response to CRT. Pre-treatment chest CT scan showed left suprahilar lung mass and mediastinal adenopathy (in blue with white arrow). Post-treatment chest CT showed NED. **(B)** Patient 22_MSK: ctDNA increase after SBRT correlated with PD on imaging. Pre-treatment CT chest showed right middle lobe lung nodule (blue and indicated by white arrow), which was treated with SBRT. Post-SBRT CT scan showed new consolidative opacities (indicated by orange arrow) in the right lower lung lobe consistent with post-radiation changes. PET/CT after treatment showed metastatic disease involving the bone and cervical lymph nodes (white arrows). **(C)** Patient 10_MSK: ctDNA clearance after CRT correlated with NED. ctDNA detection during follow-up period preceded radiographic disease recurrence by 5.3 months. The pre-treatment CT scan showed a very low volume disease at baseline (in blue with white arrow), which cleared after CRT. The subsequent radiographic imaging (image showed NED in the initially involved areas of the lung not shown) showed new subcentimeter lung nodules. After the initial recurrence (lung nodules), the following radiographic imaging showed new brain metastases (white arrows), which corresponded to an increase in ctDNA levels. Abbreviations: ctDNA, circulating tumor DNA; CRT, chemoradiation; CT, computed tomography; SBRT, stereotactic body radiotherapy; PET, positron emission tomography; PD, progressive disease; RT, radiotherapy; NED, no evidence of disease; MTM, mean tumor molecules.
