# Supplementary figures and images for: ctDNA-based detection of molecular residual disease in stage I-III non-small cell lung cancer patients treated with definitive radiotherapy
Source: Front Oncol. 2023 Sep 19;13:1253629. doi: 10.3389/fonc.2023.1253629 (PMC10546425; doi:10.3389/fonc.2023.1253629)

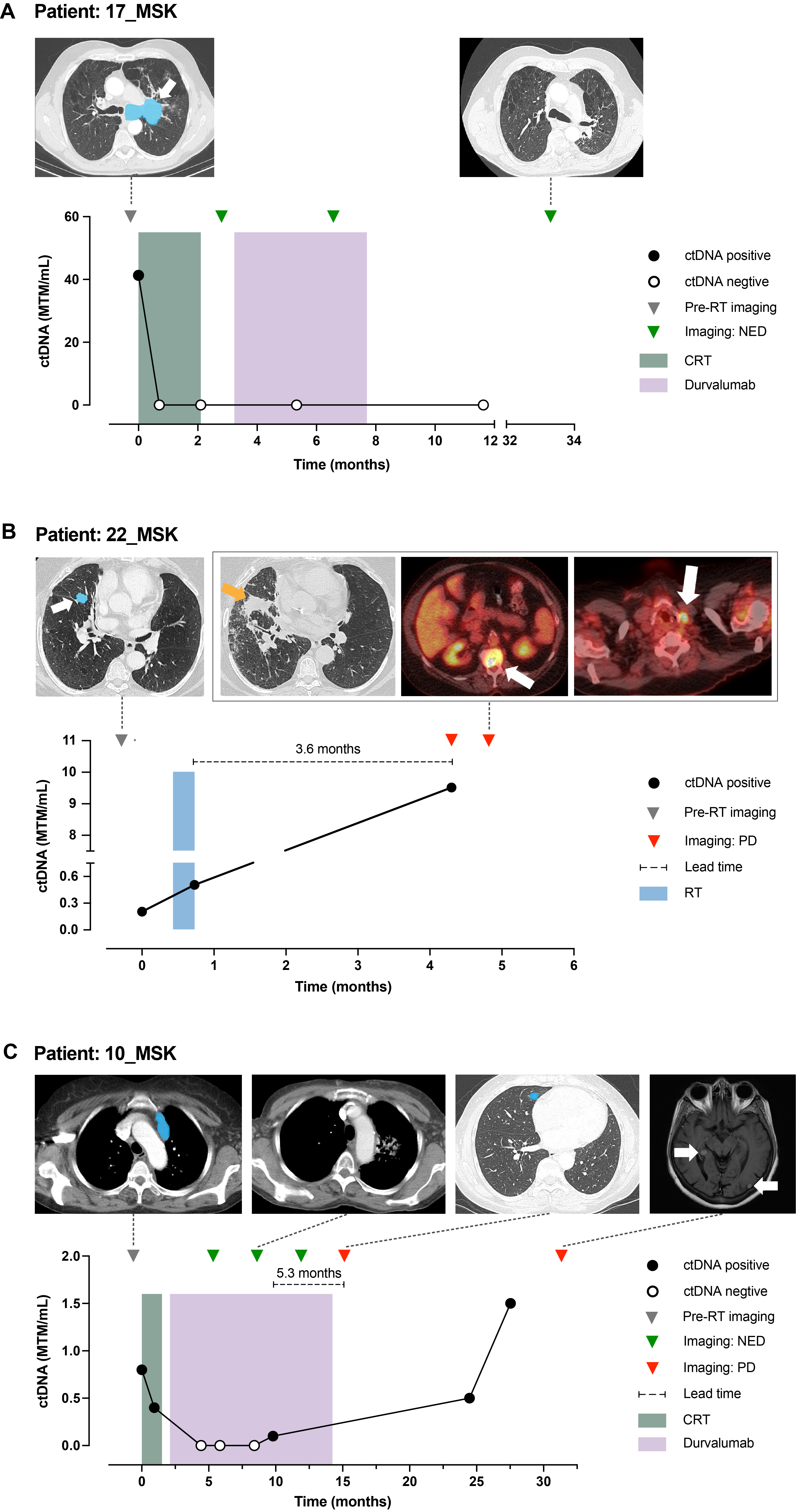

Supplement: Supplementary Figure 1 — Patient-specific changes in ctDNA levels in response to treatment and radiographic imaging data. (A) Patient 17_MSK: ctDNA clearance indicated response to CRT. Pre-treatment chest CT scan showed left suprahilar lung mass and mediastinal adenopathy (in blue with white arrow). Post-treatment chest CT showed NED. (B) Patient 22_MSK: ctDNA increase after SBRT correlated with PD on imaging. Pre-treatment CT chest showed right middle lobe lung nodule (blue and indicated by white arrow), which was treated with SBRT. Post-SBRT CT scan showed new consolidative opacities (indicated by orange arrow) in the right lower lung lobe consistent with post-radiation changes. PET/CT after treatment showed metastatic disease involving the bone and cervical lymph nodes (white arrows). (C) Patient 10_MSK: ctDNA clearance after CRT correlated with NED. ctDNA detection during follow-up period preceded radiographic disease recurrence by 5.3 months. The pre-treatment CT scan showed a very low volume disease at baseline (in blue with white arrow), which cleared after CRT. The subsequent radiographic imaging (image showed NED in the initially involved areas of the lung not shown) showed new subcentimeter lung nodules. After the initial recurrence (lung nodules), the following radiographic imaging showed new brain metastases (white arrows), which corresponded to an increase in ctDNA levels. Abbreviations: ctDNA, circulating tumor DNA; CRT, chemoradiation; CT, computed tomography; SBRT, stereotactic body radiotherapy; PET, positron emission tomography; PD, progressive disease; RT, radiotherapy; NED, no evidence of disease; MTM, mean tumor molecules. [file Image_1.jpeg]
